# Neuropathic Pain Phenotype Does Not Involve the NLRP3 Inflammasome and Its End Product Interleukin-1β in the Mice Spared Nerve Injury Model

**DOI:** 10.1371/journal.pone.0133707

**Published:** 2015-07-28

**Authors:** Verdad Curto-Reyes, Guylène Kirschmann, Marie Pertin, Stephan K. Drexler, Isabelle Decosterd, Marc R. Suter

**Affiliations:** 1 Pain Center, Department of Anesthesiology, Lausanne University Hospital (CHUV) and University of Lausanne, Lausanne, Switzerland; 2 Department of Fundamental Neurosciences, University of Lausanne, Lausanne, Switzerland; 3 Department of Biochemistry, University of Lausanne, Epalinges, Switzerland; University of Sevilla, SPAIN

## Abstract

The NACHT, LRR and PYD domains-containing protein 3 (NLRP3) inflammasome is one of the main sources of interleukin-1β (IL-1β) and is involved in several inflammatory-related pathologies. To date, its relationship with pain has not been studied in depth. The aim of our study was to elucidate the role of NLRP3 inflammasome and IL-1β production on neuropathic pain. Results showed that basal pain sensitivity is unaltered in NLRP3^-/-^ mice as well as responses to formalin test. Spared nerve injury (SNI) surgery induced the development of mechanical allodynia and thermal hyperalgesia in a similar way in both genotypes and did not modify mRNA levels of the NLRP3 inflammasome components in the spinal cord. Intrathecal lipopolysaccharide (LPS) injection increases apoptosis-associated speck like protein (ASC), caspase-1 and IL-1β expression in both wildtype and NLRP3^-/-^ mice. Those data suggest that NLRP3 is not involved in neuropathic pain and also that other sources of IL-1β are implicated in neuroinflammatory responses induced by LPS.

## Introduction

The inflammasomes are cytosolic protein complexes which act as intracellular sensors of disruption of homeostasis. Their stimulation leads to the proteolytic cleavage of the proinflammatory cytokines pro-IL-1β (interleukin-1 beta) and pro-IL-18 (interleukin-18) through the activation of caspase-1. The active complex consists of a central scaffold protein for which it is named (*e*.*g*. NLRP1, NLRP3, NLRP6, NLRC4, AIM2), an adaptor apoptosis-associated speck-like protein (ASC) containing a caspase activation and recruitment domain (CARD), which is mandatory for most inflammasomes, and the precursor form of caspase-1 enzyme, pro-caspase-1. How the inflammasomes are activated is still debated, but in the case of NLRP3 (NACHT, LRR and PYD domains-containing protein 3), one of the most studied caspase-1 activators, several signals related to cell damage and stress (generation of extracellular ATP, production of reactive oxygen species (ROS), activators that form crystalline/particulate ligands) are likely involved [1]. When the inflammasome is activated, pro-caspase-1 undergoes an autolysis leading to the formation of caspase-1, which in turn cleaves pro-IL-1β, generating the mature cytokine, which is subsequently released along with caspase-1 [2], via non-classical secretory pathways [3, 4]. The NLRP3 inflammasome is the most studied inflammasome in the central nervous system [5]. It has been linked to acute disorders (from infections [6] to acute brain injury [7]) and chronic diseases exhibiting an inflammatory component (experimental autoimmune encephalitis [8], Parkinson’s disease [9], Alzheimer’s disease [10], prion disease [11], etc.). NLRP3 was recently also implicated in fibromyalgia [12]. NLRP3 inflammasome has been studied in microglia and macrophages but has also been suggested to have functions in neurons [6, 13]. In the spinal cord, the expression of NLRP3 is increased in an experimental autoimmune encephalitis model and NLRP3 knockout mice show a delayed course of a less severe disease [14].

Following peripheral nerve injury, extensive inflammatory changes have been described in the central nervous system, which participate in the painful behaviour [15, 16]; this encompasses microglial and astrocytic reactivity and the involvement of multiple cytokines. Prototypic pro-inflammatory cytokines such as IL-1β, interleukin-6 or tumour necrosis factor-α are often cited as major contributors in the neuro-glial crosstalk during inflammatory reactions of the central nervous system [16].

A major behavioural consequence of peripheral nerve injury is pain. Pain relationship with IL-1β has been studied for a long time. IL-1β causes mechanical and thermal hyperalgesia when injected into peripheral and central tissues [17–22], and increased IL-1β expression in the spinal cord, DRG, and injured nerve in some animal models of neuropathic pain has been described [23–28].

Here we hypothesize NLRP3 inflammasome has a role in acute and chronic pain following peripheral nerve injury, as well as a role in IL-1β expression. The behavioural response and expression of the NLRP3 complex components were assessed after a nerve injury or after intrathecal injection of lipopolysaccharide (LPS). We demonstrate that pain-related behaviour in naive and neuropathic animals is unchanged in NLRP3 deficient mice and that IL-1β does not play a major role in the spinal cord in the spared nerve injury model in mice. Moreover, IL-1β expression following intrathecal LPS is independent of NLRP3.

## Methods

### Animals and surgery

All experiments were approved by the Committee on Animal Experimentation of the Canton de Vaud, Lausanne, Switzerland, in accordance with Swiss Federal law on animal care, the guidelines of the International Association for the Study of Pain (IASP) and the ARRIVE guideline (S1 Text) [29]. Mice were housed 5/cage at constant temperature (21 ± 2°C) and a 12/12 dark/light cycle. Animals had ad libitum access to water and food.

#### NLRP3^-/-^ mice

C57Bl/6-TgH(NLRP3)12Siec mice were kindly provided by the laboratory of late J Tschopp (Biochemistry Institute, University of Lausanne, Switzerland) [30, 31]. Adult male and female knockout mice or their wildtype littermates were used. Mutant and wildtype genotypes were confirmed using PCR and standard agarose gel electrophoresis.

#### Spared Nerve Injury (SNI)

Mice were randomly separated in two groups to undergo SNI or sham procedure. Animals were anaesthetised using isoflurane 1.5–2.5% (Abott AG, Baar, ZG, Switzerland). Incision was made at mid-thigh level using the femur as a landmark and a section was made through the biceps femoris. The three peripheral branches (sural, common peroneal and tibial nerves) of the sciatic nerve were exposed. Both tibial and common peroneal nerves were ligated using a 6.0 silk suture and transected. The sural nerve was carefully preserved by avoiding any nerve stretch or nerve contact [32, 33]. For animals undergoing sham surgery, same procedure was performed but the nerves remained untouched. Animals were routinely observed daily for 3 days after surgery and then once a week by the experimenter. Besides observing weight, social and individual behaviour, the operated hindpaw was examined for signs of injury or autotomy. No analgesia is provided after the surgery in order to avoid interference with chronic pain mechanisms and this is in accordance with our veterinary authorization. Suffering is minimized by careful handling and increased bedding.

### Drugs and delivery

Mice were lightly restrained and were intrathecally injected into the lumbar region, between the L5 and L6 vertebrae, using a 29G 13mm needle, with LPS (2 μg dissolved in 10 μl of 0.9% NaCl, Sigma Aldrich, L-6529) or vehicle (10 μl of 0.9% NaCl). A first injection is intended to prime the immune system; a second is administered 24 hours later.

### Quantitative PCR

Animals were sacrificed by terminal anesthesia with pentobarbital. L4 and L5 spinal cords were rapidly dissected and collected in RNA-later solution (Qiagen, Basel, Switzerland). mRNA was extracted and purified with RNeasy Plus Mini kit (Qiagen) and quantified using RNA 6000 Nano Assay (Agilent Technologies AG, Basel, Switzerland). A total of 1 μg of RNA was reverse transcribed for each sample using Omniscript reverse transcriptase (Qiagen). Gene-specific mRNA analyses were performed using the iQ SYBR-green Supermix (BioRad, Reinach, Switzerland) and the iQ5 real-time PCR detection system (BioRad) with the following conditions: 3 min at 95°C and 45 cycles of 10s at 95°C and 45s at 60°C. Primer sequences are shown in Table 1. To confirm the specificity of amplification, each qPCR product was sequenced. Briefly, qPCR products were loaded on a low melt agarose gel to first confirm the size of the amplicon. Amplicons were then subcloned in pGEM-T Vector System (Promega, Madison, WI, USA), and sent for sequencing using T7 promoter (Fasteris, Geneva, Switzerland). Only reactions with the appropriate melting curves and correct size on gel migration after amplification were analyzed. All samples were run in triplicate. GAPDH and HPRT were first validated as reference gene after SNI in mice using a similar method to our previous validation in rat [34]. Relative Quantification to reference genes was performed using the delta-delta Ct method.

**Table 1:** List of primers used on qPCR experiments.

Gene	Accession number	Amplicon length	Concentration used	Sequence (5’ > 3’), Fwd and Rev	Efficiency
Caspase-1	NM_009807.2	63	200nM	CCCAAGCTTGAAAGACAAGCC	0.92
				CCTTGTTTCTCTCCACGGCAT	
IL-1β	NM_008361	119	200nM	GAAGTTGACGGACCCCAAAA	0.85
				GCCTGCCTGAAGCTCTTGTT	
GAPDH	NM_008084.2	70	200nM	TCCATGACAACTTTGGCATTG	0.85
				CAGTCTTCTGGGTGGCAGTGA	
HPRT	NM_013556.2	96	200nM	ACTGGAAAGAATGTCTTGATTGTTG	1
				CATTTTGGGGCTGTACTGCTT	
ASC	NM_023258.4	149	200nM	CTGCAGATGGACGCCATAGAT	1.02
				GCTCCAGACTCTTCTTTAGTCGT	
				GCTCCAGACTCTTCTTTAGTCGT	

### IL-1β ELISA

Protein concentration of all samples was determined using Bradford method (Biorad). Forty to 60 μg of protein were used for ELISA analysis using the Mouse IL-1β ELISA Ready-SET-Go! eBioscience kit. Kit-supplied standards and test samples were run in duplicate following the manufacturer instructions. Samples were read at 450nm with wavelength correction at 540nm.

### Behavioural testing

Animals were tested in a temperature stable room during the light period of their day/night cycle (7h00-19h00), at the same time every day after a period of habituation to the experimental handling. They were allowed to accustom for a period of 15–30 minutes before each testing. All behavioural testing was carried out blind to treatment, and genotype.

#### Von Frey test

Animals were placed in a polymethyl methacrylate box with a wire grid floor. Withdrawal thresholds were assessed using calibrated von Frey hairs (Ugo Basile) according to the “up-down” method [35]. The 50% withdrawal threshold (in grams) was calculated using the method described by Dixon [36].

#### Hargreaves test

The noxious heat threshold of the hind paw was determined using Hargreaves plantar test [37]. Animals were placed in acrylic cubicles (8 x 5 x 10 cm) atop a uniform glass surface, and allowed to habituate before testing. An infrared light source was directed to the plantar surface of the hind paw, and the latency of withdrawal was recorded. To prevent tissue injury, the maximum stimulus duration was 20 seconds. Three responses were recorded for each hind paw, and an average response for each was taken.

#### Hot-plate test

The hot-plate assay was conducted by placing the animals on the hot-plate surface set at defined temperatures (49°C, 52°C, and 55°C). The latency of response (in seconds) until a hind paw lick or jump was determined. The cutoff was adjusted for each temperature to avoid tissue damage (60 seconds for 49°C, 30 seconds for 52°C, and 20 seconds for 55°C).

#### Acetone test

Response to cold stimulus was evaluated by applying an acetone drop formed with a syringe connected to a thin polyethylene tube to the plantar skin of animal’s hindpaw. The acetone drop was applied twice with an interval of at least one minute to each animal hindpaw and the duration of the response (licking, biting, shacking) during one minute was recorded.

#### Tail-flick assay

The tail-flick assay was conducted using a tail-flick analgesia meter (Columbus Instruments), and the mice were gently restrained in a conical plastic cloth. The latency of response (in seconds) was recorded at 2 different light beam intensities 4 and 7 (AU).

#### Pincher test

The pincher test consists of a pair of large blunt forceps (15 cm long; flat contact area: 7 mm × 1.5 mm with smooth edges) equipped with 2 strain gauges connected to a modified electronic dynamometer (Bioseb). The tips of the forceps were placed around the tail or the paw of the tested mouse, and the force applied was incremented by hand until a withdrawal response occurred. The measurement was repeated 3 times, and the mean force (in grams) that induced withdrawal was calculated.

#### Formalin test

The formalin test was conducted by injecting 10 μl of 5% formalin subcutaneously into the left hindpaw. The time (in seconds) the animal spent lifting, flinching and licking its paw was recorded at 5-minute intervals for 60 minutes.

### Statistical analysis

All data are represented as mean ± SD. ELISA, qPCR data were analysed using one-way ANOVA followed by Dunnett's post-test. Behavioural data were analysed using two-way repeated measures ANOVA followed by Bonferroni post-test where appropriate. A *P* value less than 0.05 was considered significant. All analyses were carried out using the statistical package SigmaPlot for Windows, version 12 or GraphPad Prism, version 5.03. For the IL-1 β ELISA experiment, a power study was performed from the time course data to compare sham vs SNI-1 week animals (alpha error of 0.05, power of 0.8) which yielded to n = 17 animals per group. Sample size calculation was done with GPower software [38].

## Results

### NLRP3-/- mice show no modification in pain behaviour

We first determined if the lack of NLRP3 component of the inflammasome plays any role on basal sensitivity. The lack of expression of NLRP3 gene does not modify responses to sensory stimuli in behavioural tests (see Table 2 and S1 Fig). These results show that NLRP3 does not play any role in the perception of painful stimuli in naive animals. In the absence of stimuli, inflammasome is not activated and its components are not oligomerized, which can explain the lack of changes in basal conditions of animals lacking NLRP3 expression. The absence of phenotype before challenging the system has been previously demonstrated in a different paradigm [39].

**Table 2:** Basal sensitivity of NLRP3-deficient mice.

**Mechanical sensitivity**			
Withdrawal threshold (g)	Wt	NLRP3^-/-^	p-value
von Frey	1.77 ± 0.74	1.26 ± 0.58	0.080
Pincher Tail	334.5 ± 93.0	342.2 ± 57.2	0.826
**Thermal sensitivity- Heat**			
Withdrawal latency (s)	Wt	NLRP3^-/-^	p-value
Radiant heat	7.85 ± 2.85	8.08 ± 3.33	0.872
Hot-plate– 55°C	6.36 ± 1.93	5.78 ± 1.21	0.425
Hot-plate– 52°C	11.94 ± 2.54	10.26 ± 1.63	0.095
Hot-plate– 49°C	19.03 ± 4.66	15.44 ± 3.14	0.058
Tail-flick—Intensity 4	44.48 ± 13.07	34.25 ± 19.06	0.178
Tail-flick—Intensity 7	14.96 ± 2.90	13.63 ± 4.09	0.412
**Thermal sensitivity- Cold**			
Withdrawal duration (s)	Wt	NLRP3^-/-^	p-value
Acetone test	2.3 ± 3.95	1.52 ± 0.56	0.567

### SNI and formalin-elicited pain responses do not require NLRP3 expression

**Fig 1:**
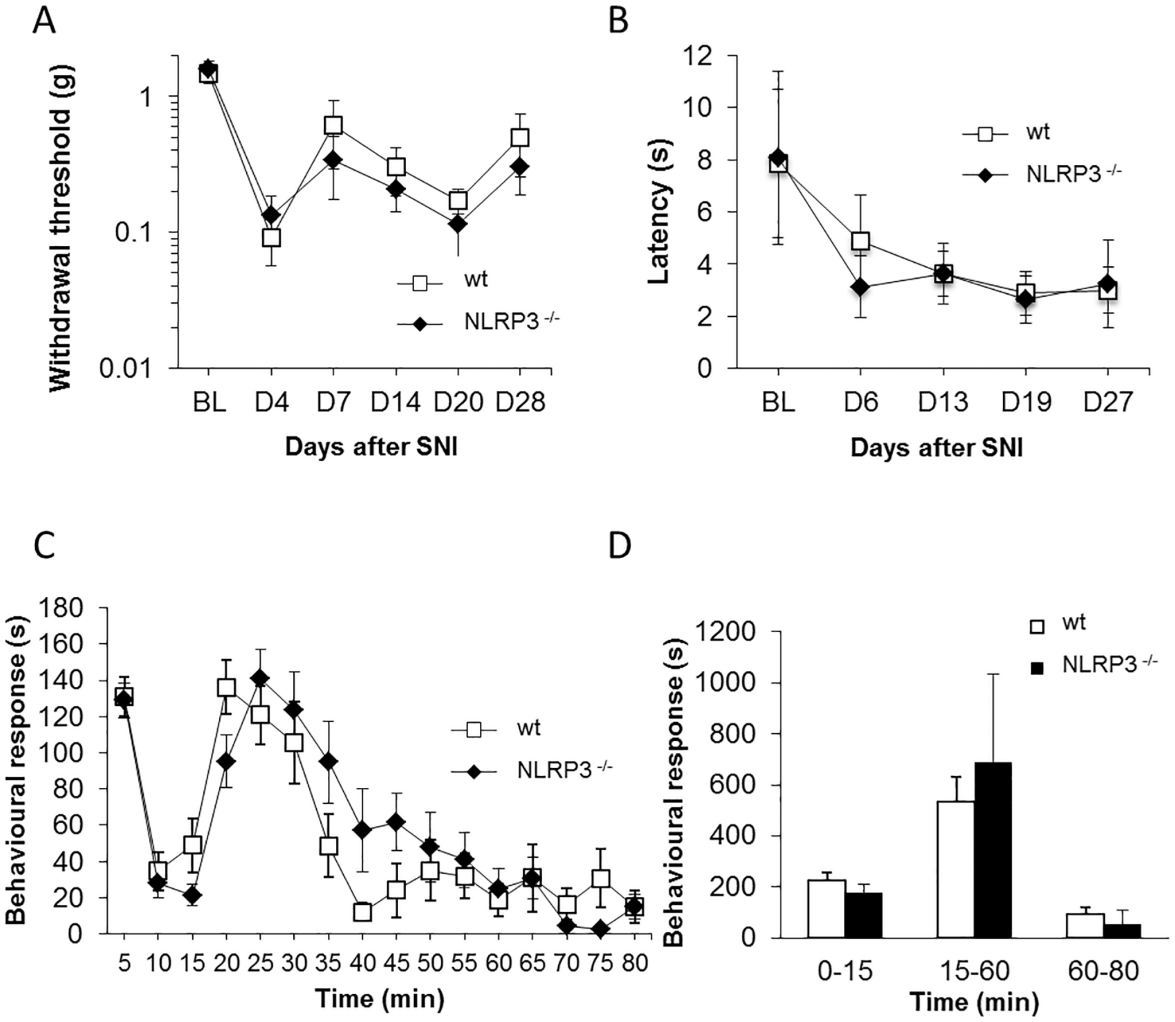
Pain development in wildtype and NLRP3^-/-^ mice after SNI. Timecourse of the development of mechanical allodynia, measured by von Frey test (A) and thermal hyperalgesia measured by Hargreaves test (B) after SNI surgery in NLRP3-deficient animals and their littermate controls. Both genotypes develop painful behaviour. No difference was observed between genotypes. Acute response to formalin injection in the hindpaw of NLRP3-deficient mice or littermate controls (C, D). Timecourse of the nocifensive response did not reveal difference between genotypes during the first or second phase of the test. P ≥ 0.05, ANOVA two-way with Bonferroni correction. Data are expressed as mean ± SD, n = 10 animals per group for all panels.

We then tested how the animals’ sensitivity is altered when challenged by a neuropathic or an inflammatory injury. NLRP3-deficient and wildtype mice underwent SNI and behavioural tests were performed to assess the expected development of mechanical allodynia and thermal hyperalgesia in the injured limb [32]. Results of a light mechanical stimulus (von Frey test) show that both wildtype and knockout animals develop allodynia 7 days after SNI surgery, evidenced by a lowering of the response threshold. This symptom remains constant in both genotypes until 28 days after SNI (Fig 1A). The application of radiant heat on the plantar surface of the affected limb showed thermal hyperalgesia, evidenced by a shortening of withdrawal latency, without difference between genotypes (Fig 1B).

Mechanical allodynia and thermal hyperalgesia are not affected by NLRP3 deletion, which suggest that this inflammasome is not necessary for the development of neuropathic pain related to peripheral nerve injury.

We then tested the possible involvement of NLRP3 inflammasome in the responses following intraplantar formalin injection leading to acute inflammatory pain behaviour. The administration of 10 μl of 5% formalin elicited the classical biphasic response with a first phase, corresponding to the direct stimulation of nociceptors, and a second phase, in which inflammatory and central sensitization mechanisms are involved. Both phases were similar in NLRP3^-/-^ mice and wildtype controls (Fig 1C and 1D).

### Inflammasome complex do not change after SNI surgery

**Fig 2:**
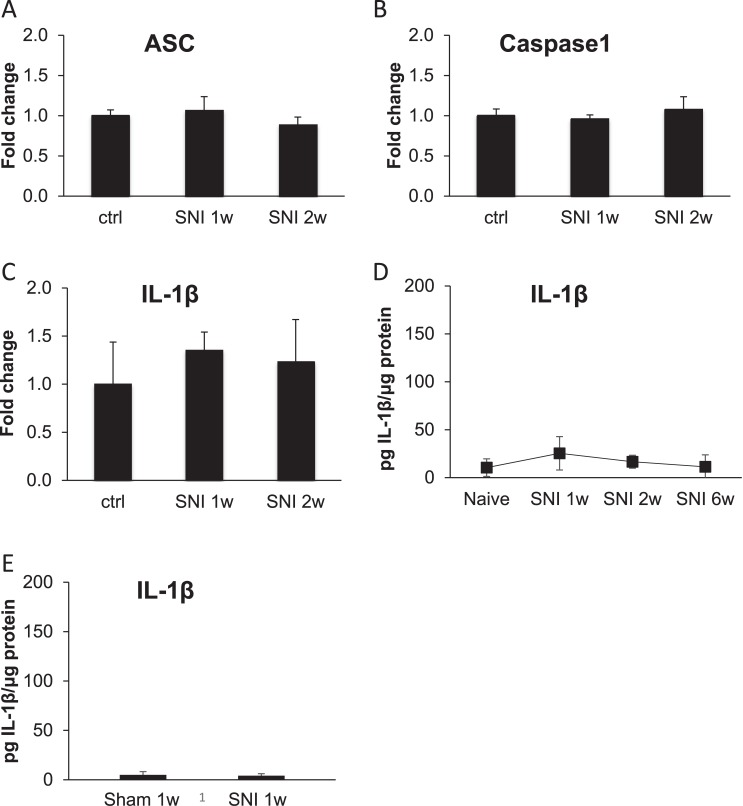
Levels of NLRP3 components in spinal cord after SNI. mRNA levels of ASC (A), caspase-1 (B) and IL-1β (C) in spinal cord of mice following SNI measured by qPCR. IL-1β protein levels in SNI surgery-bearing mice (D, E) measured by ELISA. No difference was observed between timepoints. P ≥ 0.05, ANOVA with Dunnett’s correction or Student’s t-test (E). Data are expressed as mean ± SD, n = 3–5 animals per group for panels A-D and n = 17 for panel E.

In the absence of behavioural phenotype in naive animals but also in neuropathic and inflammatory pain models, we investigated the reactivity of inflammasome components and IL-1β. Expression of inflammasome components in the spinal cord one and two weeks after SNI revealed that mRNA levels of ASC, caspase-1 and IL-1β assessed by qPCR remained unaltered (Fig 2A–2C). There is no regulation at the protein level of IL-1β 1, 2 and 6 weeks after SNI compared to naive animals (Fig 2D), and between sham and SNI operated animal at 1 week (Fig 2E). With these results we can conclude that NLRP3 inflammasome complex in the spinal cord is not implicated in the neuropathic pain responses in the SNI model, neither is IL-1β.

### Intrathecal LPS injection elicits changes in NLRP3 inflammasome complex

**Fig 3:**
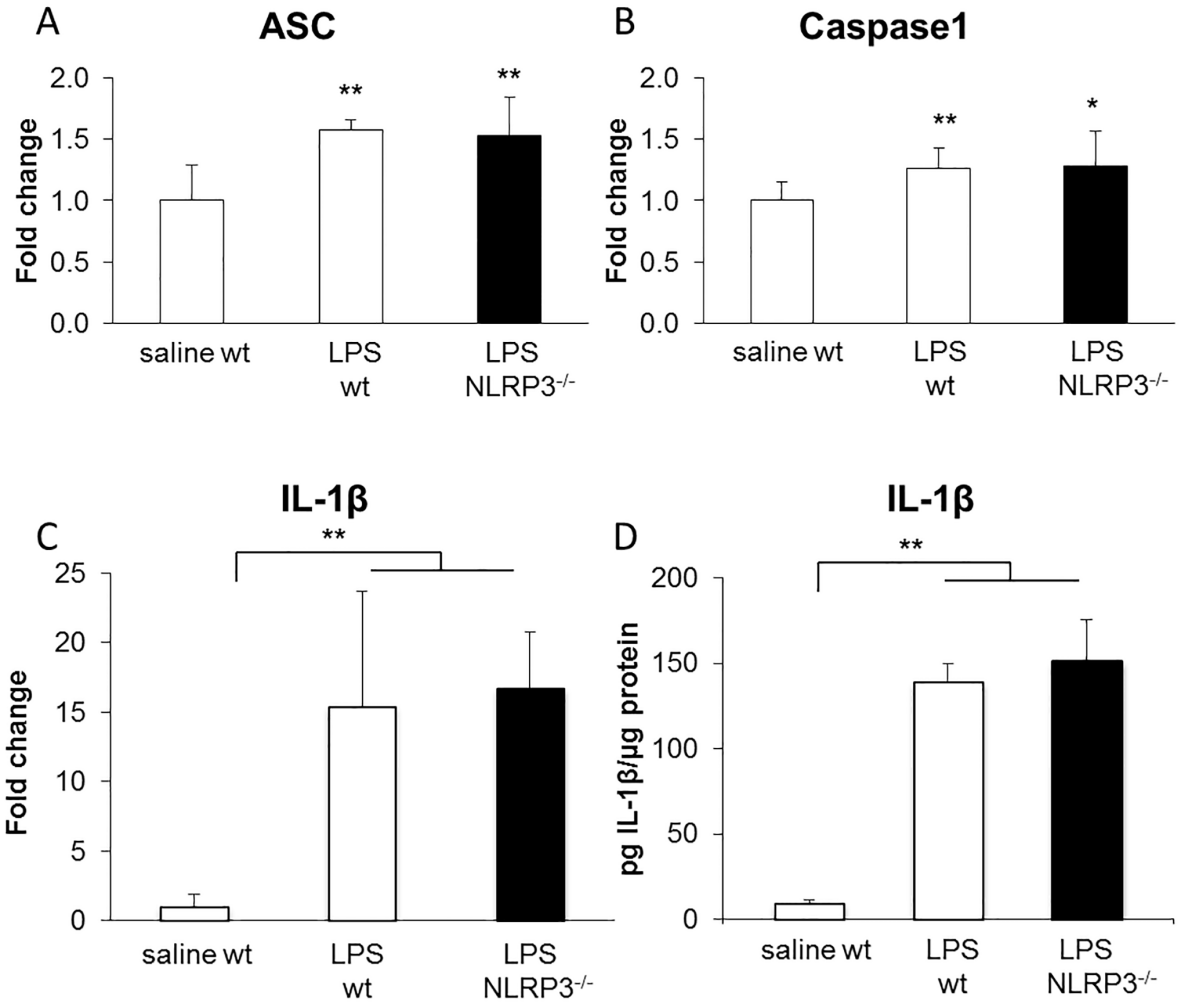
Levels of NLRP3 components in spinal cord after intrathecal LPS injection. mRNA levels measured by qPCR of ASC (A), caspase-1 (B) and IL-1β (C) in spinal cord of NLRP3-deficient mice or littermate controls injected intrathecally with LPS or vehicle. IL-1β protein levels in transgenic or control mice injected with either vehicle or LPS (D) measured by ELISA. Differences were observed between vehicle and LPS-injected animals (*) P ≤ 0.05, (**) P ≤ 0.01, ANOVA with Dunnett’s correction. Data are expressed as mean ± SD, n = 4–7 animals per group for all panels.

To validate our negative results regarding NLRP3 involvement in neuropathic pain, we investigated the inflammasome component in a central inflammatory insult. We used the intrathecal administration of LPS model which leads to hyperalgesia [40–42] and to IL-1β production on spinal cord slice preparation [42]. We designed these experiments as positive controls, in order to confirm that the lack of regulation of IL-1β after SNI was not an experimental artefact. Wildtype and NLRP3^-/-^ mice were given 2 intrathecal doses of LPS (2 μg) or vehicle, in a 24 hours interval [42]. mRNA expression of ASC, caspase-1 and IL-1β significantly increased after intrathecal LPS injection in wildtype and mutant mice. This increase is not statistically different in NLRP3^-/-^ mice when compared with wildtype controls (Fig 3A–3C). Similarly, protein levels of IL-1β are upregulated after intrathecal injection of LPS, but no difference was observable between NLRP3^-/-^ mice and wildtype controls (Fig 3D).

## Discussion

The main objective of our work has been to elucidate the participation of the NLRP3 inflammasome on the development and maintenance of neuropathic pain. We have used total knockout of NLRP3 in a model of peripheral nerve injury (SNI) and a model of acute and delayed inflammatory pain. Our results showed that NLRP3 is not involved in basal sensitivity to innocuous and painful stimuli, nor in formalin-induced pain responses or SNI pain-related behaviour. We then observed that the elements of NLRP3 complex and its final product, IL-1β, were not regulated in this model of nerve-injury pain. This unexpected negative result made us control our methodology using a model of neuroinflammation (intrathecal LPS administration) in which we witnessed a large increase in IL-1β. The increase was however similar in WT and NLRP3-KO mice.

Our working hypothesis of the NLRP3-IL-1β pathway involvement in pain models was based on previous literature implicating IL-1β in pain. Briefly, IL-1β was first shown to induce pain related behaviour when exogenously administered systemically [43], intraneurally in the sciatic nerve [44] or intrathecally [21, 45]. Mechanistically the superfusion of IL-1β on organotypic culture of spinal cord slices induces increase in excitatory post-synaptic potentials (EPSP) [46]. In rats, an increase of IL-1β at mRNA or protein level was observed after spinal nerve injury [25, 26, 47], after hind paw and thoracic incisions [48], spinal cord injury [49], zymosan or formalin injection [50] and in a cancer pain model [51]. In mice, an increase in IL-1β in the spinal cord was observed following spinal cord injury [52], sciatic nerve injury [24] and in cancer pain models [53] and also in the sciatic nerve in the partial sciatic nerve injury [54].

Our lack of regulation of IL-1β in the spinal cord in the SNI model of mice is surprising. Differences are well-known between species [55] and most abovementioned experiments were performed in rats. In mice, Da Silva et al. used a partial ligation of the sciatic nerve model in mice and observed an increase in IL-1β in the spinal cord 7 days after the injury, the same timepoint as our study [24]. Besides they use a different strain, their model exhibits a different anatomical relationship between injured and spared nerves from ours [56]. In partial sciatic nerve ligation, distal injured branches undergoing Wallerian degeneration are in contact with the intact ones, partly responsible for the pain development, something that does not happen in ours [33, 57].

The intrathecal LPS injection was performed as positive control for IL-1β increase and indeed IL-1β was highly upregulated. The similar increase of IL-1β in mice lacking NLRP3 as in wildtype animals suggests other sources for this interleukin. These could be the activation of ASC-independent inflammasomes such as NLRC4 and NLRP1, that interact and activate directly pro-caspase-1 [58, 59], caspase-8, which is activated after the stimulation of Toll-like receptors 3 and 4 [60] or also other neutrophil-derived proteases such as cathepsin G, proteinase 3 and elastase [61]. In the context of neuropathic pain, metalloproteinases have been described as a source of IL-1β for the induction and maintenance of hypersensitivity after SNL but no increase was observed in the spinal cord after the nerve injury [62]. In a peripheral inflammatory pain model, NLRC4 and ASC but not NLRP3 were implicated in IL-1β increase and behavioural responses [63].

To summarize, our findings show that neither NLRP3 nor IL-1β are implicated in the SNI model of neuropathic pain. To discard the possibility of a technical problem, we show an increase of IL-1β in an inflammatory model of spinal cord, and validated our qPCR results by sequencing. We have also demonstrated that intrathecal LPS-derived IL-1β increase is NLRP3 independent and we hypothesize that other sources of this interleukin are implicated.

## Supporting Information

S1 Text ARRIVE guidelines. (PDF)

S1 Fig Basal sensitivity of NLRP3-deficient mice. (PDF)
