# Supplementary material for: Neuropathic Pain Phenotype Does Not Involve the NLRP3 Inflammasome and Its End Product Interleukin-1β in the Mice Spared Nerve Injury Model
Source: PLoS One. 2015 Jul 28;10(7):e0133707. doi: 10.1371/journal.pone.0133707 (PMC4517753; doi:10.1371/journal.pone.0133707)
Supplement: S1 Fig — (PDF) [file pone.0133707.s002.pdf]

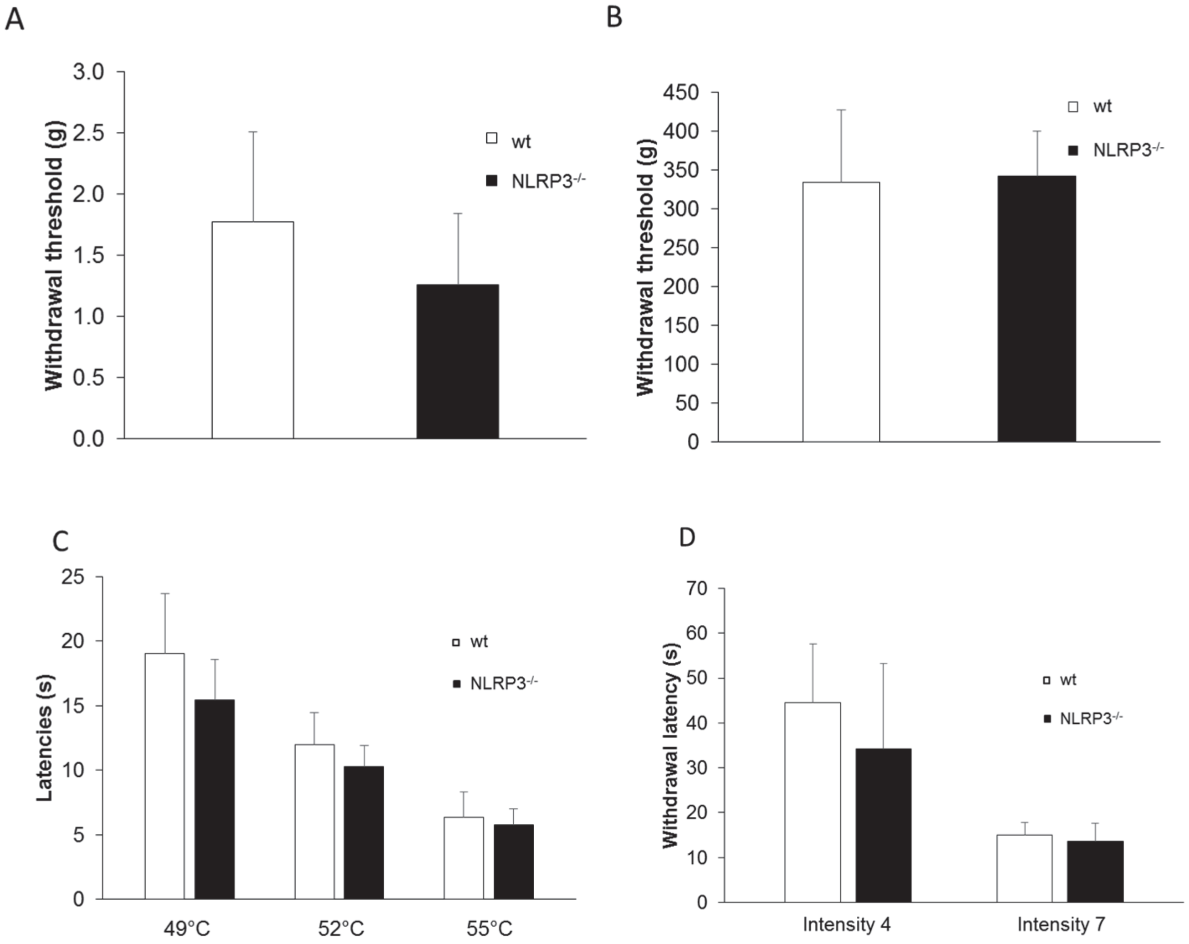

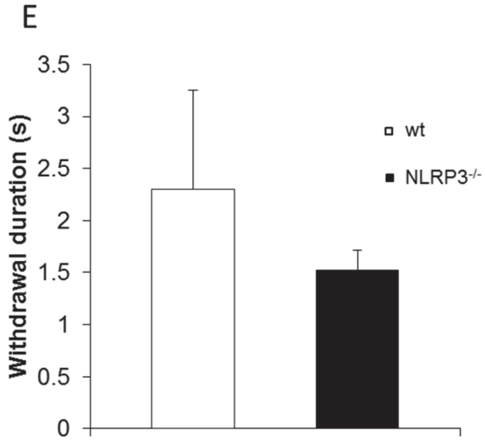

Supplemental figure legend.:

A) Withdrawal threshold upon mechanical stimulation with von Frey filaments
B) Tail pincher: Withdrawal upon pinch of the tail
C) Hot plate stimulation, latency of paw withdrawal upon stimulation at 49°, 52° and 55°
D) Tail flick: Tail withdrawal upon heat stimulation
E) Withdrawal duration after stimulation with acetone

N=10/group, $p>0.05$ for all WT vs NLRP3$^{-/-}$
